# Spatio-temporal patterns of bladder cancer incidence in Utah (1973-2004) and their association with the presence of toxic release inventory sites

**DOI:** 10.1186/1476-072X-10-16

**Published:** 2011-02-28

**Authors:** Léa Fortunato, Juan José Abellan, Linda Beale, Sam LeFevre, Sylvia Richardson

**Affiliations:** 1 Department of Epidemiology and Biostatistics and MRC-HPA Centre for Environment and Health, Imperial College London, London, UK; 2 Centre for Public Health Research (CSISP), Valencia, Spain; 3 CIBER Epidemiología y Salud Pública (CIBERESP), Valencia, Spain; 4 Environmental Epidemiology Program, Office of Epidemiology, Utah Department of Health, Salt Lake City, Utah, US

## Abstract

**Background:**

The authors analyse the spatio-temporal variations of the incidence of bladder cancer between 1973 and 2004 in Utah at the census tract level (496 areas) to highlight areas of high and low relative risks that remained so throughout the 32 year period. Using these identified areas, a novel strategy is used to carry out a geographical case-control study of association between the risk of bladder cancer and presence of Toxic Release Inventory sites, where areas with stable high RRs are 'case areas' and all remaining areas with stable non increased risks are 'control areas'.

**Results:**

The time trend of bladder cancer risk fluctuated over the study period: A steady decrease was observed, followed by an abrupt increase from 1992 to 2004. Using a Bayesian space-time model, 93 census tracts were classified as having an excess relative risk and 81 a lower relative risk, sustained over the 32 years. We showed that these high relative risk areas for bladder cancer were associated with the presence of Toxic Release Inventory sites, after adjusting for the proportion of Latter-Day Saint Church members as an area level proxy for smoking habits.

**Conclusions:**

Our study has demonstrated that the modeling of data in time and space has additional benefits over a purely spatial analysis. In addition to highlighting the areas with high and low relative risks, this model also allows the simultaneous study of persistency of spatial patterns over time and detection of 'unusual' time trends that may warrant further investigation.

## Introduction

Bladder cancer (ICD10 C67) accounts for approximately two-thirds of all urinary tract cancers and is the ninth most common cancer worldwide [1]. In the US, bladder cancer is the fourth most frequent tumor in men and the eighth in women [2]. US time trends for the period 1975-2004 showed an initial increase (1975-1987) followed by stable rates from 1987 to 2004 [3].

Tobacco smoking and occupational exposure to aromatic amines are the two major established risk factors for bladder cancer [4-6]; age, gender and dietary habits have been shown to influence bladder cancer risk. Environmental factors have also been linked to the risk of bladder cancer [7], especially exposure to chemical components, such as volatile organic compounds (VOC), released in the air by industrial processes [8]. In the US, facilities that release chemicals must report annually their toxic release inventory (TRI) information to the US Environmental Protection Agency (EPA) and to the State in which they are located [9]. The database contains detailed information on nearly 650 chemicals and chemical categories for 23,000 industrial and other facilities since 1988, based on their acute or chronic effects on human health or the environment. A positive association between VOC emissions from TRI sites and urinary cancer risk in Indiana was found: approximately 8.70% of the variation in urinary cancer among Indiana counties could be explained by VOC emissions [8].

In the state of Utah, the 2000-2004 age-adjusted incidence rate of bladder cancer was 16.6 per 100,000 people, 29.4 per 100,000 men and 6.5 per 100,000 women [10]. For the same period, the national average was 37.3 per 100,000 men and 9.4 per 100,000 women [3]. Utah has long been recognized for its low incidence of bladder and other cancers, attributable primarily to low prevalence of smoking and alcohol consumption among members of the Church of Latter-day Saints (LDS) [11]. The high percentage of LDS members in the Utah population (about 65% in 2000) reduces the impact of these two risk factors. This makes Utah an ideal area in which to study environmental exposures, and more specifically the potential risks associated with the presence of TRI sites. Indeed, adjusting for strong individual level confounders such as alcohol consumption is difficult in geographical studies. Three population-based studies linked Utah Cancer registry data with LDS Church membership records to obtain site-specific cancer incidence for LDS and non-LDS populations in Utah from 1967-75 [12], 1971-85 [13] and 1995-1999 [14], and found lower incidence rates for cancers among LDS males and females, compared with non-LDS groups. For the period 1995-99, the age-adjusted (2000 US standard population) incidence rate for bladder cancer was 15.5 (LDS), 23.9 (non-LDS) and 18.0 (Utah) per 100,000 men, and 3.3 (LDS), 8.0 (non-LDS) and 4.5 (Utah) per 100,000 women. In general air emissions from Utah TRI sites have declined. Some of this decline can be referred to changes of the industrial environment of Utah as well as changes in the efficiency of processes and pollution control. This decline also reflects changes in reporting requirements implemented by EPA several times during the history of TRI.

In collaboration with the US Centers for Disease Control and Prevention and the Utah Department of Health (UDOH), we investigated the spatial and temporal distribution of bladder cancer incidence in Utah from 1973-2004. The aim was to study the persistence of spatial patterns and to highlight any unusual time patterns in localized areas. We also investigated the link between spatial and temporal variations of bladder cancer and TRI sites. We used a class of Bayesian spatio-temporal models that decompose the risk variability into spatial and temporal main effects plus space-time interactions [15-18]. Recently Abellan et al. [19] suggested a Bayesian hierarchical model formulation, within a Binomial framework, in which the stable spatial and time patterns as well as departures from these stable components were characterized and estimated simultaneously. We built on this work to develop a new analysis strategy for geographical study of environmental exposure based on (i) characterizing areas that exhibit stable relative risks (RR) and (ii) conducting a 'geographical case-control study' by comparing relevant indicators of environmental exposure between high RR areas and the others, adjusting for known confounders whenever possible. We report results on the risk of bladder cancer associated with exposure to TRI sites, using the proportion of LDS as proxy for smoking.

## Methods

### Data sources

**Figure 1:**
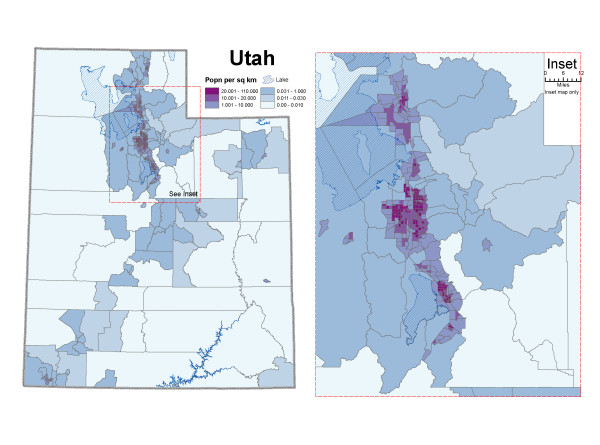
**Population per square kilometer by census tract**. Figure 1 displays the population density in the 496 census tracts in Utah, based on 2000 U.S census. Utah's population was mainly concentrated in the counties of Weber, Davis, Salt Lake and Utah.

Incident cases of bladder cancer and population estimates at the census tract level from 1973-2004 were obtained from the UDOH. The state of Utah is divided into 29 counties and 496 census tracts. According to the 2000 US census, Utah's population was mainly concentrated in the centre of the state: counties of Weber, Davis, Salt Lake and Utah (Figure 1). The statewide population density ranged from 0.0 to 110.0 inhabitants per square kilometer (median = 10.0, interquartile range = 1.4-19.9).

Over the 32-year period, there were a total of 5,882 incident cases of bladder cancer (4,560 males and 1,322 females) in Utah. To reduce the level of sparseness while keeping the time dimension, data were aggregated over 8 four-year periods, 1973-1976,..., 2001-2004 for the analysis. The average number of observed cases per census tract per 4-year period was 1.5 (1.2 male and 0.3 female) and the median was 1.1 (0.6 male and 0 female). The expected cases were calculated on the basis of average age-specific incidence rates for the study region and the total period 1973-2004 (median = 1.3 per census tract per 4-year period, interquartile range = 0.7-2.0, Table 1).

**Table 1:** Distribution of observed and expected numbers of incident cases of bladder cancer, Utah, 1973-2004

Period	Min	1st Quartile	Median	Mean	3rd Quartile	Max
	Observed numbers of incident cases of bladder cancer					

1973-1976	0.00	0.00	0.00	0.89	1.00	8.00
1977-1980	0.00	0.00	1.00	1.11	2.00	9.00
1981-1984	0.00	0.00	1.00	1.13	2.00	8.00
1985-1988	0.00	0.00	1.00	1.24	2.00	14.00
1989-1992	0.00	0.00	1.00	1.35	2.00	11.00
1993-1996	0.00	0.00	1.00	1.79	3.00	10.00
1997-2000	0.00	1.00	2.00	2.16	3.00	15.00
2001-2004	0.00	1.00	2.00	2.19	3.00	12.00
**Average**	**0.0**	**0.3**	**1.1**	**1.5**	**2.3**	**10.9**

	Expected numbers of incident cases of bladder cancer					

1973-1976	0.00	0.30	0.66	0.92	1.32	4.65
1977-1980	0.00	0.40	0.80	1.04	1.47	4.66
1981-1984	1.19 × 10^-5^	0.49	0.93	1.18	1.72	4.65
1985-1988	7.14 × 10^-5^	0.58	1.13	1.35	1.90	5.22
1989-1992	1.07 × 10^-4^	0.70	1.32	1.54	2.16	5.89
1993-1996	8.09 × 10^-6^	0.90	1.57	1.75	2.36	6.28
1997-2000	0.00	1.11	1.73	1.98	2.58	6.89
2001-2004	3.50 × 10^-5^	1.26	1.93	2.20	2.87	8.55
**Average**	**0.0**	**0.7**	**1.3**	**1.5**	**2.0**	**5.8**

We used data from the US EPA's Toxic Release Inventory [9]. In Utah, 355 TRI sites located in 126 census tracts reported chemical releases between 1988 and 2004. Sixty-three TRI sites were located in 22 census tracts and reported emissions for 10 or more consecutive years.

Information about the fraction of the population belonging to the LDS Church in each county was provided by Membership and Statistical Records Division [20]. Data at a smaller level were not available; therefore we assumed that the distribution of the LDS members was homogeneous in a county.

### Statistical analyses

Bayesian hierarchical models have been successfully used in disease mapping with low counts of cases when the disease is rare. Different spatio-temporal models are proposed in literature according to the decomposition of the temporal and space main effects and the consideration of the space-time interactions (e.g. [15-18]). For statistical analyses of Utah data, we adapted the Bayesian hierarchical space-time model proposed by Abellan et al. [19] to the Poisson framework. The first level corresponds to the usual Poisson likelihood. The relative risks (RR) are then treated as random effects at the second level and given a specific space-time decomposition including space-time interactions.

At the first level, the number of cases *O_ij _*for census tract *i *= 1,..., 496 and time period *j *= 1,..., 8 is assumed to follow a Poisson distribution with mean *E_ij_ρ_ij_*, *E_ij _*and *ρ_ij _*being the known expected number of cases and unknown RR in area *i *and period *j*, respectively. At the second level, the log-RRs are decomposed into log(*ρ_ij_*) = *α *+ *s_i _*+ *t_j _*+ *v_ij _*, with *s_i_*, *t_j _*and *v_ij _*treated as random effects. The term exp(*α*) is the overall RR of disease in the study region compared to the reference rate. The spatial effects *s_i _*represent the main spatial patterns of the RRs over the 32 years analysed. The temporal effects *t_j _*pick up the global temporal trend of the whole study region. The space-time interaction terms *v_ij _*capture any departure from the main spatial and temporal patterns. Therefore, these parameters may be used to characterise the stability of the underlying spatial patterns, with large fluctuations of {*v_ij_*, *j *= 1,..., 8} indicating 'unusual' temporal risk trends in area *i*. We refer to time trends that depart from the global one, though not necessarily clustered geographically.

As is usual in the Bayesian paradigm, prior distributions are assigned to the model parameters and the random effects. For the spatial main effects *s_i_*, we chose the usual BYM model [21] corresponding to the sum of two random effects, one spatially structured and one unstructured to account for both types of variation in the risks. For the time effects *t_j _*we used a first order random walk. With this prior, the time effect of period *j *is smoothed towards its temporal neighbors *j *- 1 and *j *+ 1. For the space-time interactions, Abellan et al. [19] suggested using a mixture with two distributions: the first one models small values of the *v_ij _*that reflect only residual noise, whereas the second is specifically introduced to capture substantial departures from the space and time main effects that indicate instability. Specifically, we considered: $v_{ij}\sim \pi \text{Normal}(0,\sigma_{1}^{2})+(1-\pi )\text{Normal}(0,\sigma_{2}^{2})$. Both components have Normal distributions with mean 0 and variances $\sigma_{1}^{2}$ and $\sigma_{2}^{2}$, respectively, with $\sigma_{1}^{2}$ assumed to be small. To insure identification of both components, we used the re-parametrisation proposed by Robert [22], which assumes $\sigma_{2}^{2}=\sigma_{1}^{2}+\kappa ,\kappa >0$. The prior for *π *is uniform on 0[1]. We used WinBUGS software [23] to obtain a sample from the posterior distribution of the model parameters.

To tease out 'stable' additive space plus time patterns from 'unusual' space-time interactions we built a classification rule based on the posterior probabilities {*p_ij_*} that each space-time interaction parameter *v_ij _*comes from the second distribution of the mixture, following Abellan et al. [19]. High values of *p_ij _*would be indicative of the corresponding *v_ij _*being large, hence indicating a clear departure from main space and time effects. Typically if *p_ij _*> 0.5, we would consider the corresponding *v_ij _*to be large. This cut-off value of 0.5 has to be taken as a guideline and higher values are typically preferred so as to be more specific and better isolate the unusual space-time patterns, particularly in cases of sparse data. In this study, we used the decision rule: an area *i *is 'unusual' if *p_ij _*> 0.6 for at least one period *j*. To compute these probabilities we introduced latent allocation variables *z_ij _*that take the value 1 or 2 if *v_ij _*comes from the first component of the mixture or from the second one, respectively; therefore *p_ij _*= *P *(*z_ij _*= 2| data). To highlight the areas with increased spatial RR, we used a rule proposed by Richardson et al. [24] based on the posterior probability of excess RR: a census tract *i *is classified as having an excess spatial RR if *P*(exp(*s_i_*) > 1 | data) > 0.8. Using a simulation study, they showed that this rule has good sensitivity and specificity properties and represents weighted trade-offs between false-positive results (i.e. declaring an area as having elevated risk when in fact its underlying true risk equals the background level) and false-negative results (i.e. declaring an area to be in the background when in facts its underlying risk is above the background level). Clearly, before using a cut-off rule, it is advisable to refer to simulation studies with a similar range of expected counts where the rule has been calibrated as well as carry out a sensitivity study as in [25,26]. Note that even if we are in a spatio-temporal context, the decision rule to classify areas with high spatial RR is based only on the spatial random effects exp(*s_i_*) and not on the space-time random effects exp(*s_i _*+ *t_j _*+ *v_ij _*). Indeed as clearly showed in [27], including the time trend component to highlight elevated spatial risks could give undue difference to the time trends and would require calibrating the decision rules for each period.

To assess the association between the risk of bladder cancer incidence and environmental hazards, we performed a geographical case-control study. The idea was to use stable spatial patterns to analyse this association, while the data on exposure are not available for the whole study period. In the case of large number of space-time departures, assuming stable exposure might not be relevant, leading to a misinterpretation of the results. We considered areas with stable high RR (as defined above) as 'case areas' and all remaining areas with stable non increased risks as 'control areas'. We further classified areas as exposed if they had at least one active TRI site for 10 or more years during the period 1988-2004. The rationale for this is that if environmental exposure from TRI sites has an effect in bladder cancer, it is likely that the association is the result of a long latency period. We then computed the odds ratio for 'exposure' and its associated 95% confidence interval (95% CI). We performed this analysis with and without adjustment for the proportion of LDS members in each area, as a proxy for smoking habits.

## Results

### Spatial and temporal main effects

**Figure 2:**
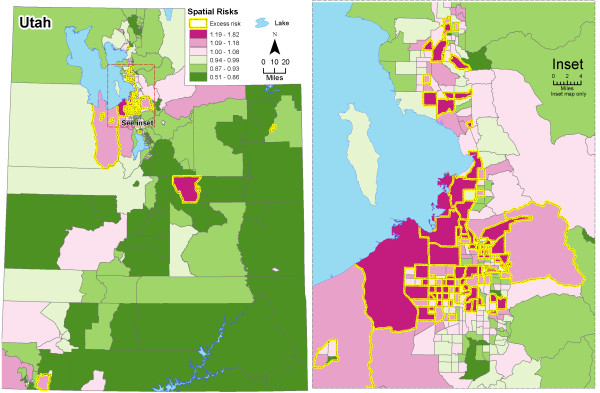
**Posterior medians of spatial relative risks, both genders**. Figure 2 displays the posterior medians of spatial relative risks exp(*s_i_*) using the spatio-temporal model. The areas with yellow borders are classified as having an elevated risk using the rule. *P*(exp(*s_i_*) > 0.8.

**Figure 3:**
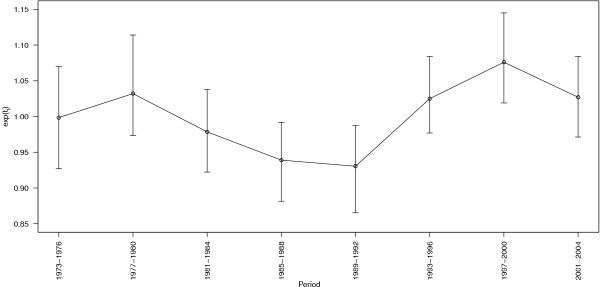
**Posterior median and 95% confidence interval of main temporal trends in each period, both genders**. Figure 3 displays the posterior median (dots) and 95% confidence interval (vertical bars) of main temporal trends exp(*t_j_*) in each period.

The map of the estimated spatial RRs (Figure 2) shows evidence of spatial heterogeneity with higher RRs in central areas around Salt Lake City. These should be interpreted as the estimated average risk for each area over the 32 years analyzed. To better highlight the areas having an excess RR, in Figure 2 we over impressed the boundaries in yellow denoting the areas with posterior probabilities *P*(exp(*s_i_*) > 1|data) above 0.8. Using that rule, 96 out of the 496 (19.4%) areas were considered as having an excess RR. Of those, 90 (93.8%) were located in the centre of Utah (Figure 2 right) and 72 (75.0%) belonged to Salt Lake county. The 'significant' high RRs ranged between 1.14 and 1.82 (interquartile range = 1.20-1.31), reflecting that the rule is not a repetition of the RR map but integrates the size of expected numbers and the spatial structure. Using a similar rule for the detection of areas with a low RR (i.e. *P*(exp(*s_i_*) > 1|data) < 0.2), we obtained that 82 out of the 496 (16.5%) were considered as such. Contrary to the areas with an excess RR, those were not spatially clustered. When we used a more restrictive decision rule, i.e. with a cut-off probability of 0.9 (instead of 0.8), we found 51 and 32 census tracts with high and low RRs, respectively. Figure 3 shows the estimated main temporal trends for each period (posterior median and 95% credibility interval). Overall, risks were higher at the beginning and at the end of the 32-year period. We observed a slow but continuous decrease in time trends between the periods 1977-1980 and 1989-1992, followed by a steep increase until the period 1997-2000. The RRs in periods 1985-1988 and 1989-1992 were significantly lower, around -6.1% and -7.0%, respectively. The period 1997-2000 showed a significant excess RR around 7.6%.

The maps of the smoothed RRs for each period are provided in Additional files section [see Additional files 1, 2, 3, 4, 5, 6, 7, 8], showing overall good stability.

### 'Unusual' patterns

**Figure 4:**
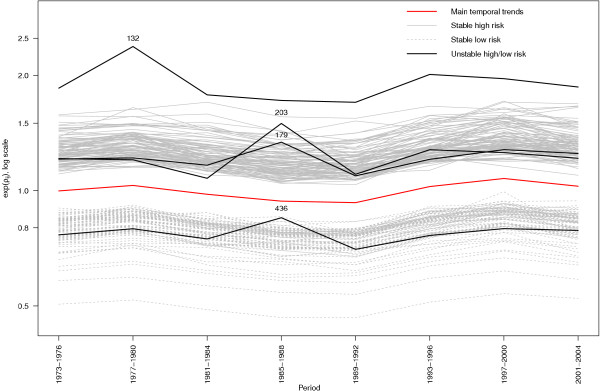
**Posterior medians of the relative risks in each period, both genders**. Figure 4 displays the posterior medians of relative risks exp(*ρ_ij_*) in each period. Three areas (ID 132, 179, 203) out of the 96 high risk areas have unusual temporal patterns. One area (ID 436) out of the 82 low risk areas has an unusual temporal patterns.

Using the decision rule *p_ij _*> 0.6 for at least one *j*, we found 13 (2.6%) census tracts with an 'unusual' temporal trend, which were not geographically clustered. This cut-off isolated the tail of the empirical distribution of space-time interactions (not shown). As a consequence, the space-time patterns of these 13 areas (Figure 4 left) were different from the rest which were almost rather flat (Figure 4 right).

### Areas with stable high/low RR

**Figure 5:**
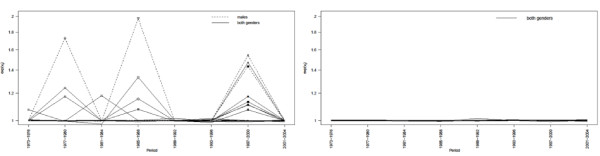
**Estimated space-time interactions, both genders**. Figure 5 displays the estimated space-time interactions *v_ij _*for the 13 'unusual' areas according the rule *p_ij _*> 0.6 (left) and 13 randomly selected 'stable' areas (right). The symbols show the common 'unusual' areas for males only and both genders.

Combining the rules used previously for declaring areas with 'significantly' high RR and 'stable' patterns, we found that in 93 out of the 96 areas with increased spatial RR, this excess risk was persistent over time. In the remaining 3 areas (with ID 132, 179 and 203; Figure 5), the RR was high on average, but it was not stable. Among the 82 areas with low RR, 81 were considered as stable.

### Patterns for males only

The same analyses were carried out for males which represent 77.5% of overall incidence. Overall, the patterns were similar as those found for both genders. We found that 86 areas out of 496 were considered as having an excess spatial RR with the same spatial distribution observed for both genders. Compared with the results for both genders, the persistent high RRs were shrunk (from 1.12 to 1.47) but with a similar spatial distribution; 76 areas were common for both analyses. Among the 82 areas with low RR, 59 were in common with those found for both genders. Only 4 census tracts were considered as 'unusual'.

### Association with environmental exposure associated to TRI sites

To assess the relationship between bladder cancer risk and the presence of TRI sites in Utah's census tracts, as a proxy for environmental hazards, we performed a geographical case-control study on the stable areas comparing the proportion of areas with TRI sites between high risk areas (93) and the others (390) for both genders. Of the 22 census tracts with TRI sites over the 10 years, 21 showed a stable spatial RR. The odds ratio (OR) for presence of TRI sites was 2.73 (95% CI = 1.05-6.69, both genders). When restricted to males only, the distribution of the TRI sites was similar and the OR was 2.96 (95% CI = 1.14-7.17). This suggested a positive association between presence of TRI sites and higher risk of bladder cancer for both genders and for males only.

To control for smoking, we used the proportion of LDS members as a surrogate of smoking habits. We grouped the distribution of LDS members in two groups (based on the median of 58%). The distribution of stable census tracts according to the proportion of LDS and the presence of TRI sites is shown in Table 2. A significant negative association between the proportion of LDS and risk of bladder cancer was found (Pearson correlation coefficient -0.36, 95% CI: -0.44, -0.28). This finding was consistent with previous studies showing that bladder cancer risk is lower in areas where the percentage of LDS is higher. The OR adjusted for the proportion of LDS was 2.73 (95% CI: 1.03, 7.23) and 3.15 (95% CI: 1.16, 8.52) for both genders and males, respectively, strengthening the previous association. Using logistic regression with the proportion of LDS as a continuous variable, the OR associated with the presence of TRI sites was 2.80 (95% CI: 1.04, 7.18) and 3.26 (95% CI: 1.21, 8.37) for both genders and males, respectively.

**Table 2:** Contingency table of census tracts, according their spatial relative risks and the presence/absence of TRI sites, stratified by the proportion of LDS (proxy for smoking)

LDS	TRI sites	Persistent spatial relative risks	
		Cases	Controls
		Both genders/Males	Both genders/Males
(0.29;0.58]	Presence	6/6	6/6
	Absence	71/69	166/170

(0.58;0.88]	Presence	2/2	7/8
	Absence	14/8	211/224

Total		93/84	390/408

The same analyses were conducted using only the 82 census tracts with stable lower RR as control areas (instead of the 390 areas) for both genders. Among them, only one census tract was exposed. The ORs for both genders associated with the presence of TRI sites were 7.52 (95% CI: 1.34, 141.25) and 5.95 (95% CI: 0.99, 114.71) without and with adjustment for the proportion of LDS, respectively. Such wide confidence intervals highlighted the fact that including all the stable areas in the analysis increased the power and therefore decreased the uncertainty of the estimates.

## Discussion

We adapted the model suggested by Abellan et al. [19] in the context of binomial variability to the Poisson case in order to analyze the space-time patterns of bladder cancer incidence in Utah for the period 1973-2004. Sparseness is quite common in disease mapping at small area level. Richardson et al. [24] showed that Bayesian hierarchical models for disease mapping are highly specific but may have low sensitivity in the presence of sparseness. We aggregated the number of cases over 4-year periods to partly remedy this. The distribution of Utah population is highly heterogeneous, with nearly 77% concentrated in 4 of the 29 Utah counties (Salt Lake, Utah, Davis and Weber). As the population in these areas was relatively high, we had more statistical power to detect not only spatial excess RRs, but also sizeable space-time interactions.

Some of the risk factors for bladder cancer are different for males and females as occupational exposure is more predominant amongst males. But in our analysis, including the females enabled us to strengthen the patterns observed for males only, pointing towards non-occupational risk factors.

Census tracts are small statistical subdivisions of a county. They usually have between 2,500 and 8,000 residents. Administrative boundaries may not be ideal for mapping health outcomes and for interpreting associations between environmental exposures and health effects. This is also related to the modifiable areal unit problem, which arises from over-imposing an arbitrary spatial aggregation on a continuous spatial process [28]. Changes in spatial aggregation or arrangement of areas can affect the underlying values for those areas, so observations are usually only relevant for the scale of analysis and should not be imputed to individuals.

Our results indicated that 19.4% of census tracts had an excess RR, and most of them were located in Salt Lake County. Our model was also used to assess areas for which the excess RR was stable over time. We found that in 93 areas out of the 96 with high spatial RR, this excess was sustained over the 32 years considered. This suggests that risk factors were continually present in most areas in Utah. The performance of exceedance probabilities to detect areas with high or low risk may be dependent on the model used (i.e. whether spatially unstructured or spatially structured random effects are included, and the way the spatial structure is modelled too) and on the data as well (level of sparseness, weak or strong spatial autocorrelation, etc.). The model chosen here for the spatial random effects as well as the rules we applied to the pure spatial effects to classify areas have been thoroughly investigated in the last years. Lawson et al. [29] and Best et al. [30] carried out simulation studies to compare several models in the pure spatial context and showed that the CAR is one of the most robust models for spatial random effects in disease mapping. Recently, it has been argued that it performs poorly in the case of heavy spatial autocorrelation [31], but this is rarely the case with incidence or mortality data of chronic diseases. The exceedance probability rules, *P*(exp(*s_i_*) > 1|data) > 0.8, used here to classify the spatial risks have their limitations [25,26] but their general usefulness as a measure of uncertainty has been proved in simulation studies [24].

We also explored which areas showed 'unusual' time trends compared with statewide trends. Using the decision rule based on marginal posterior probabilities with a threshold of 0.6, we found 13 census tracts classified as 'unusual'. Space-time departures were mainly detected for the period 1997-2000 when the highest level of risk was observed in the temporal trend. This unexpected time variation in the risk could correspond to a change in recording practices or to a real excess of cases and our results deserve further scrutiny. With a long latency disease like bladder cancer, we did not expect to find a large number of significant space-time interactions that can be interpreted epidemiologically. This was consistent with our findings. These areas were not geographically clustered, which suggests that chance rather than an environmentally driven process generated these unusual area-specific patterns.

The time trend of risk of bladder cancer incidence in Utah fluctuated over the 32 years analyzed. A steady decrease was observed between 1977 and 1992, followed by an abrupt increase until the period 1997-2000. Assuming that bladder cancer has a latency of 15-30 years, then the spike in 1997-2000 may be correlated with the urbanization and industrialization of Weber, Davis, Salt Lake and Utah counties (77% of the population). Prior to 1975, these counties were primarily rural and agricultural in nature with some industries around the small urban centers in the county. From 1975 through 1985, these counties underwent a substantial urbanization as the freeway I-15 was completed in 1974 and Utah actively recruited industry to establish in these counties. These counties had substantial growth (near 35% increase in population) period between 1970 and 1980 which is nearly double that of the decade before and after (both around 17-19%). During those years, the new population was brought into close proximity with the new industry, and consequently the occupational exposure of people working in industry may be more important. Subsequent to those years, improved regulatory controls and technologies have reduced the exposure even though the population remains close to the sources.

Staying at the aggregated level, we thus proposed a novel strategy of using areas with stable risk to carry out a 'geographical' case-control study of association between risk of bladder cancer and presence of TRI sites. To this end, we considered areas with high risk sustained over time as cases, and the remaining areas with stable 'neutral' risk as controls. We restricted the case-control study to areas with sustained risk because of the mismatch between the periods of exposure and health outcomes considered here. Data on TRI sites are only available from 1988, though toxic sites may have existed long before that. The results of the case-control study showed some evidence of positive association after adjusting for the fraction of the population belonging to the LDS Church. However, because of the important migration, the association of bladder cancer with the presence of TRI sites may be diluted [32].

Because the TRI data is collected for regulatory purposes and not for exposure assessment purpose, there is considerable criticism over its use to estimate exposure. The EPA frequently changes the reporting requirements in what agents are to be reported and at what threshold (magnitude of release) they are to be reported. In addition, there is no assurance that all the industries that should be reporting are reporting. In some cases what is reported is left to the interpretation of the industry. Releases are computed using a complex modeling system rather than actual monitoring. For those industries that are reporting, in order to minimize the regulatory impacts, they might underestimate their actual release when possible. There has also been a concern about a potential problem of miss-locating the TRI sites since coordinates of these sites are only stored with minimal decimal places. We used more precise information from the UDOH data for which aerial photographs are used to pinpoint each site to remedy this.

## Conclusion

Our study confirmed that the modeling of data in time and space has additional benefits over a purely spatial analysis. In addition to highlighting the areas with high and low RR, the space-time model also allowed the simultaneous study of persistency of spatial patterns over time and of detection of 'unusual' time trends that may warrant further investigation. The accompanying strategy that we proposed to analyse the association between health outcomes and environmental risk factors pointed to an association of bladder cancer with the presence of TRI sites.

## Competing interests

The authors declare that they have no competing interests.

## Authors' contributions

LF designed and carried out the statistical analyses, and drafted the paper. JJA helped design the statistical analysed and direct its implementation. LB provided help with the mapping of the results and GIS issues. SL provided the data and helped prepare the Materials and Discussion sections of the text. SR supervised the entire study (study's analytic strategy, implementation and interpretation of the results). All authors read and approved the final manuscript.

## Supplementary Material

Additional file 1 **Figure S1: Posterior medians of relative risks for 1973-1976 (period 1), both genders**. Figure S1 displays the posterior medians of relative risks *ρ*_*i*1 _for 1973-1976, using the spatio-temporal model. Click here for file

Additional file 2 **Figure S2: Posterior medians of relative risks for 1977-1980 (period 2), both genders**. Figure S2 displays the posterior medians of relative risks *ρ*_*i*2 _for 1977-1980, using the spatio-temporal model. Click here for file

Additional file 3 **Figure S3: Posterior medians of relative risks for 1981-1984 (period 3), both genders**. Figure S3 displays the posterior medians of relative risks *ρ*_*i*3 _for 1981-1984, using the spatio-temporal model. Click here for file

Additional file 4 **Figure S4: Posterior medians of relative risks for 1985-1988 (period 4), both genders**. Figure S4 displays the posterior medians of relative risks *ρ*_*i*4 _for 1985-1988, using the spatio-temporal model. Click here for file

Additional file 5 **Figure S5: Posterior medians of relative risks for 1989-1992 (period 5), both genders**. Figure S5 displays the posterior medians of relative risks *ρ*_*i*5 _for 1989-1992, using the spatio-temporal model. Click here for file

Additional file 6 **Figure S6: Posterior medians of relative risks for 1993-1996 (period 6), both genders**. Figure S6 displays the posterior medians of relative risks *ρ*_*i*6 _for 1993-1996, using the spatio-temporal model. Click here for file

Additional file 7 **Figure S7: Posterior medians of relative risks for 1997-2000 (period 7), both genders**. Figure S7 displays the posterior medians of relative risks *ρ*_*i*7 _for 1997-2000, using the spatio-temporal model. Click here for file

Additional file 8 **Figure S8: Posterior medians of relative risks for 2001-2004 (period 8), both genders**. Figure S8 displays the posterior medians of relative risks *ρ*_*i*8 _for 2001-2004, using the spatio-temporal model. Click here for file
