# Supplementary figures and images for: Spatio-temporal patterns of bladder cancer incidence in Utah (1973-2004) and their association with the presence of toxic release inventory sites
Source: Int J Health Geogr. 2011 Feb 28;10:16. doi: 10.1186/1476-072X-10-16 (PMC3058003; doi:10.1186/1476-072X-10-16)

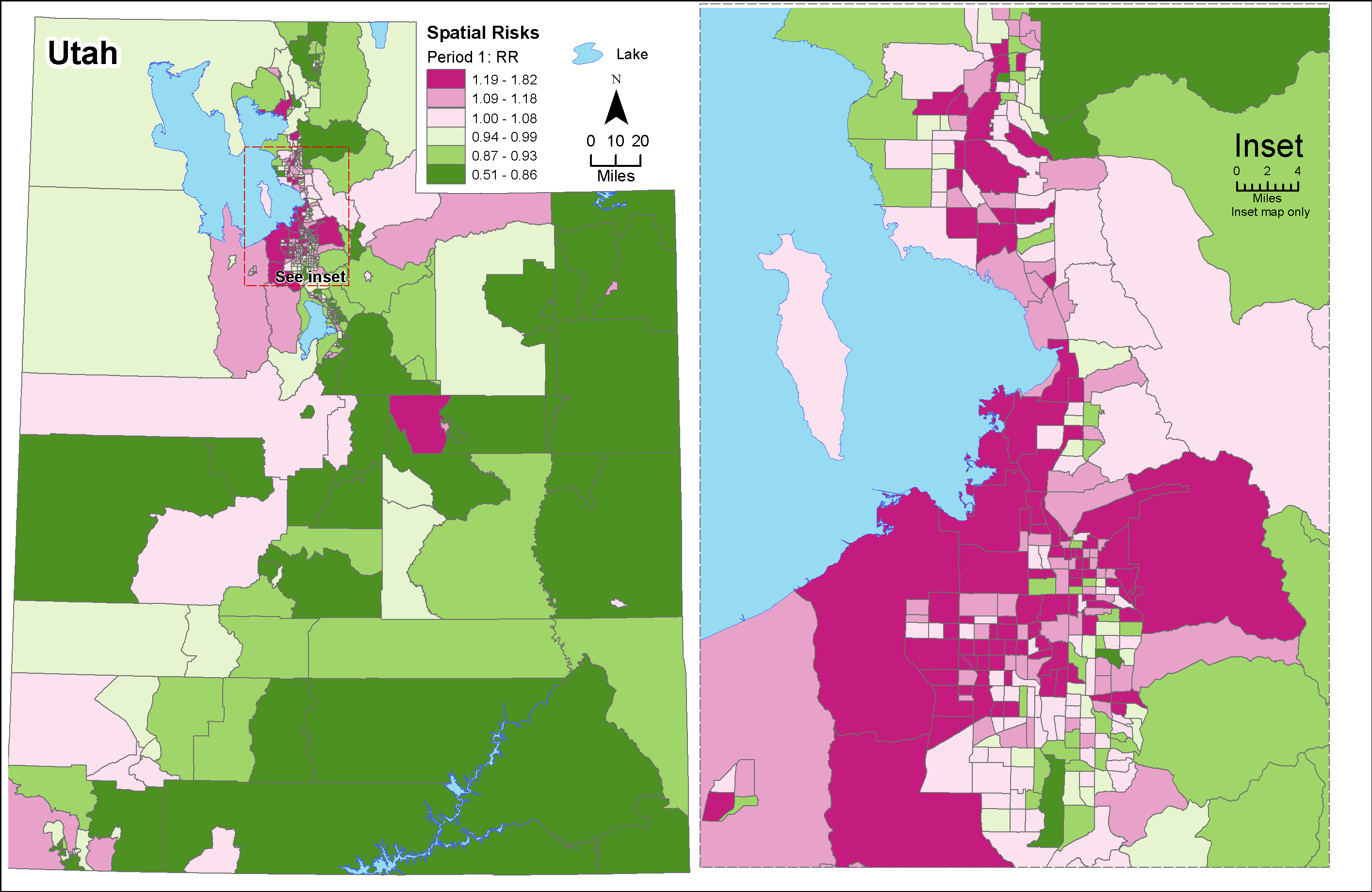

Supplement: Additional file 1 — Figure S1: Posterior medians of relative risks for 1973-1976 (period 1), both genders. Figure S1 displays the posterior medians of relative risks ρi1 for 1973-1976, using the spatio-temporal model. [file 1476-072X-10-16-S1.PNG]

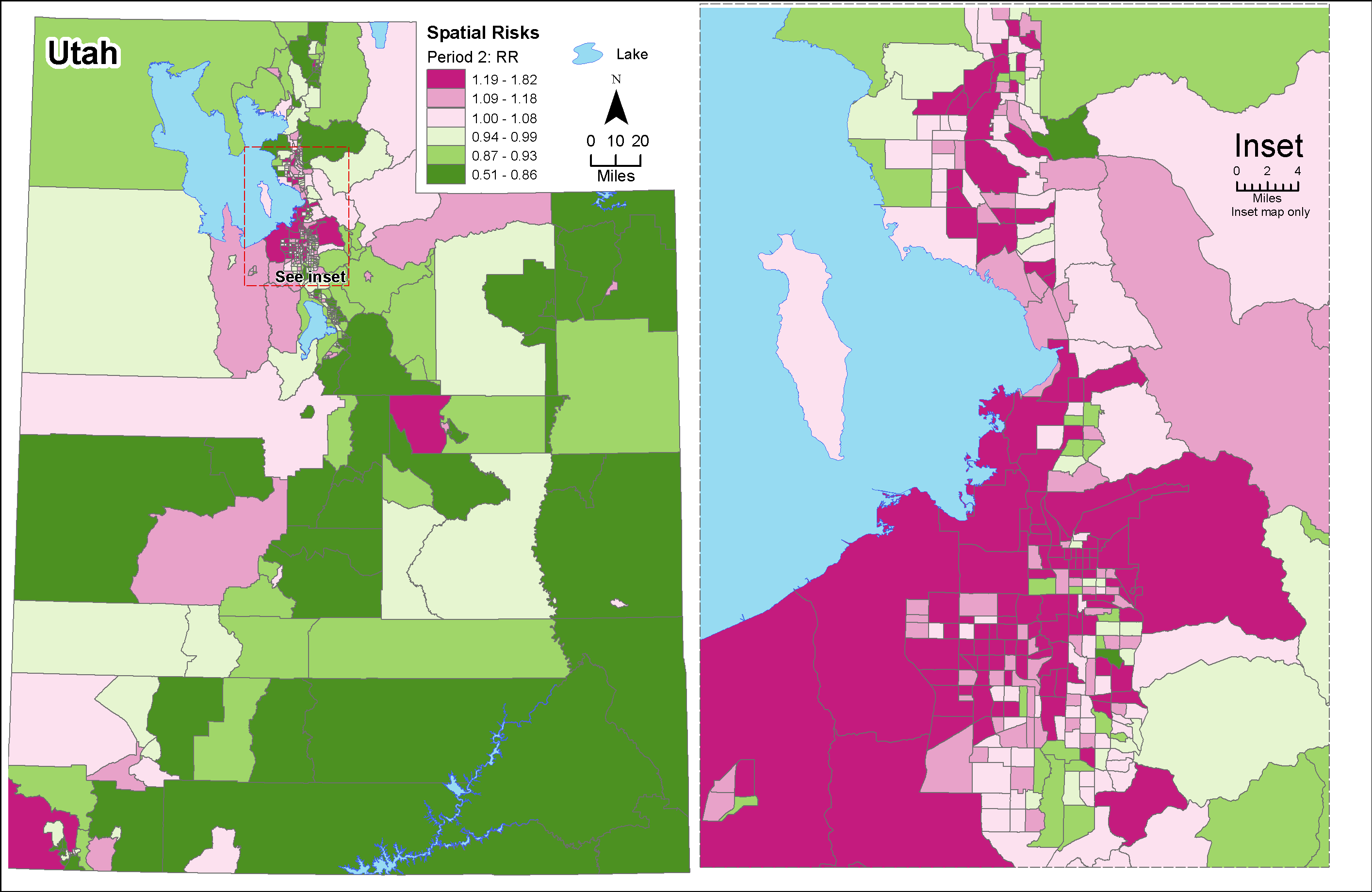

Supplement: Additional file 2 — Figure S2: Posterior medians of relative risks for 1977-1980 (period 2), both genders. Figure S2 displays the posterior medians of relative risks ρi2 for 1977-1980, using the spatio-temporal model. [file 1476-072X-10-16-S2.PNG]

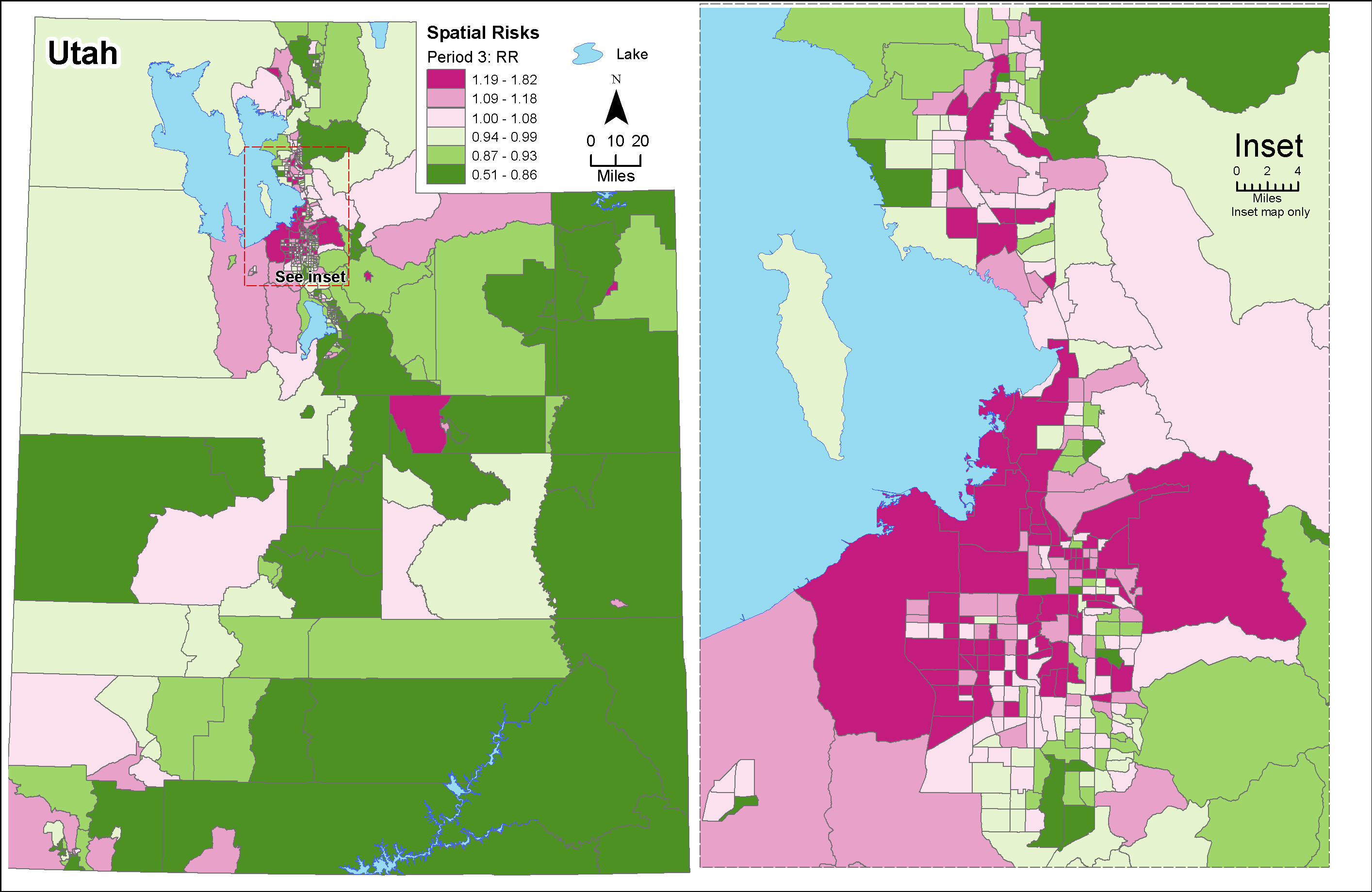

Supplement: Additional file 3 — Figure S3: Posterior medians of relative risks for 1981-1984 (period 3), both genders. Figure S3 displays the posterior medians of relative risks ρi3 for 1981-1984, using the spatio-temporal model. [file 1476-072X-10-16-S3.PNG]

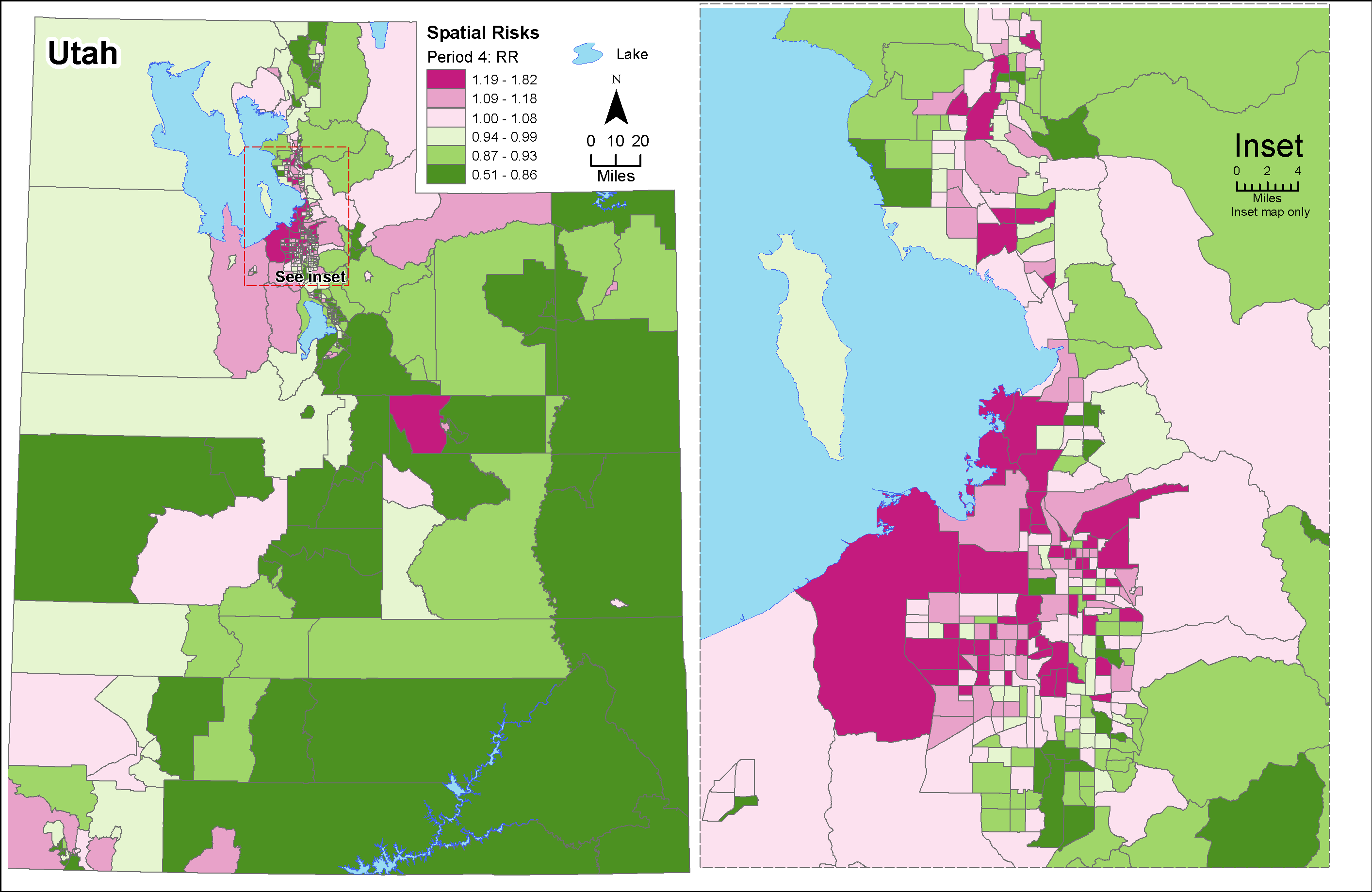

Supplement: Additional file 4 — Figure S4: Posterior medians of relative risks for 1985-1988 (period 4), both genders. Figure S4 displays the posterior medians of relative risks ρi4 for 1985-1988, using the spatio-temporal model. [file 1476-072X-10-16-S4.PNG]

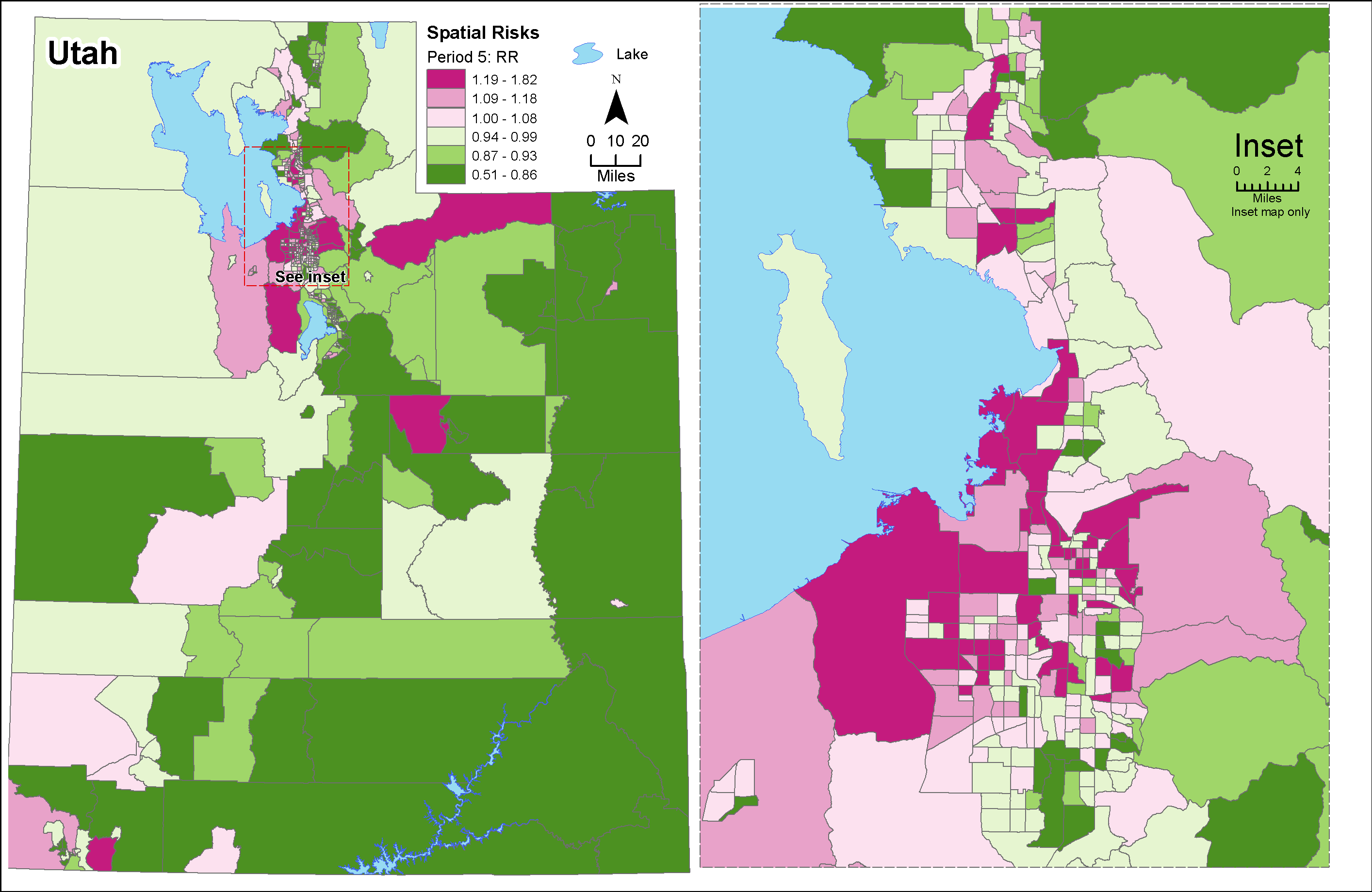

Supplement: Additional file 5 — Figure S5: Posterior medians of relative risks for 1989-1992 (period 5), both genders. Figure S5 displays the posterior medians of relative risks ρi5 for 1989-1992, using the spatio-temporal model. [file 1476-072X-10-16-S5.PNG]

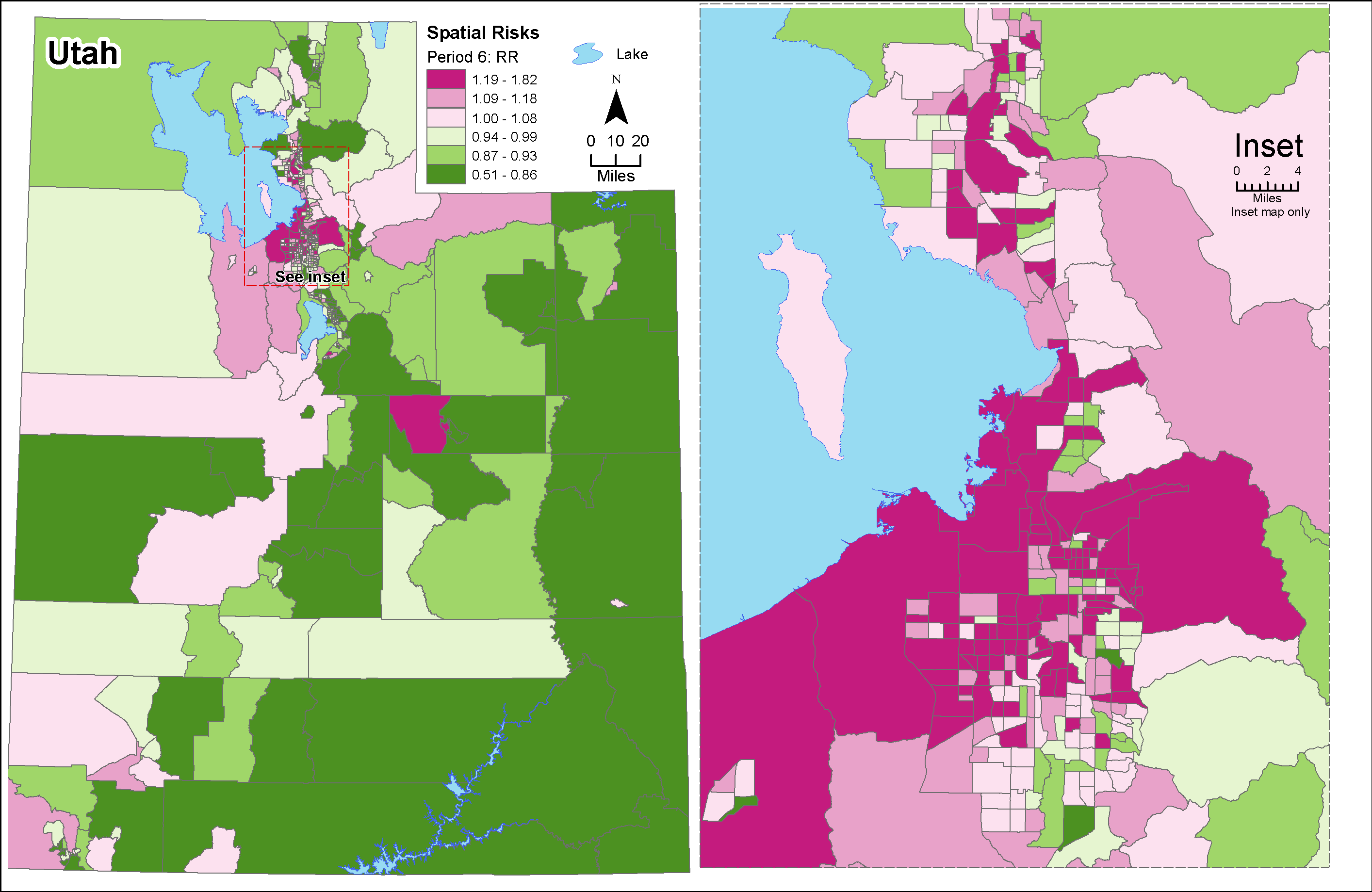

Supplement: Additional file 6 — Figure S6: Posterior medians of relative risks for 1993-1996 (period 6), both genders. Figure S6 displays the posterior medians of relative risks ρi6 for 1993-1996, using the spatio-temporal model. [file 1476-072X-10-16-S6.PNG]

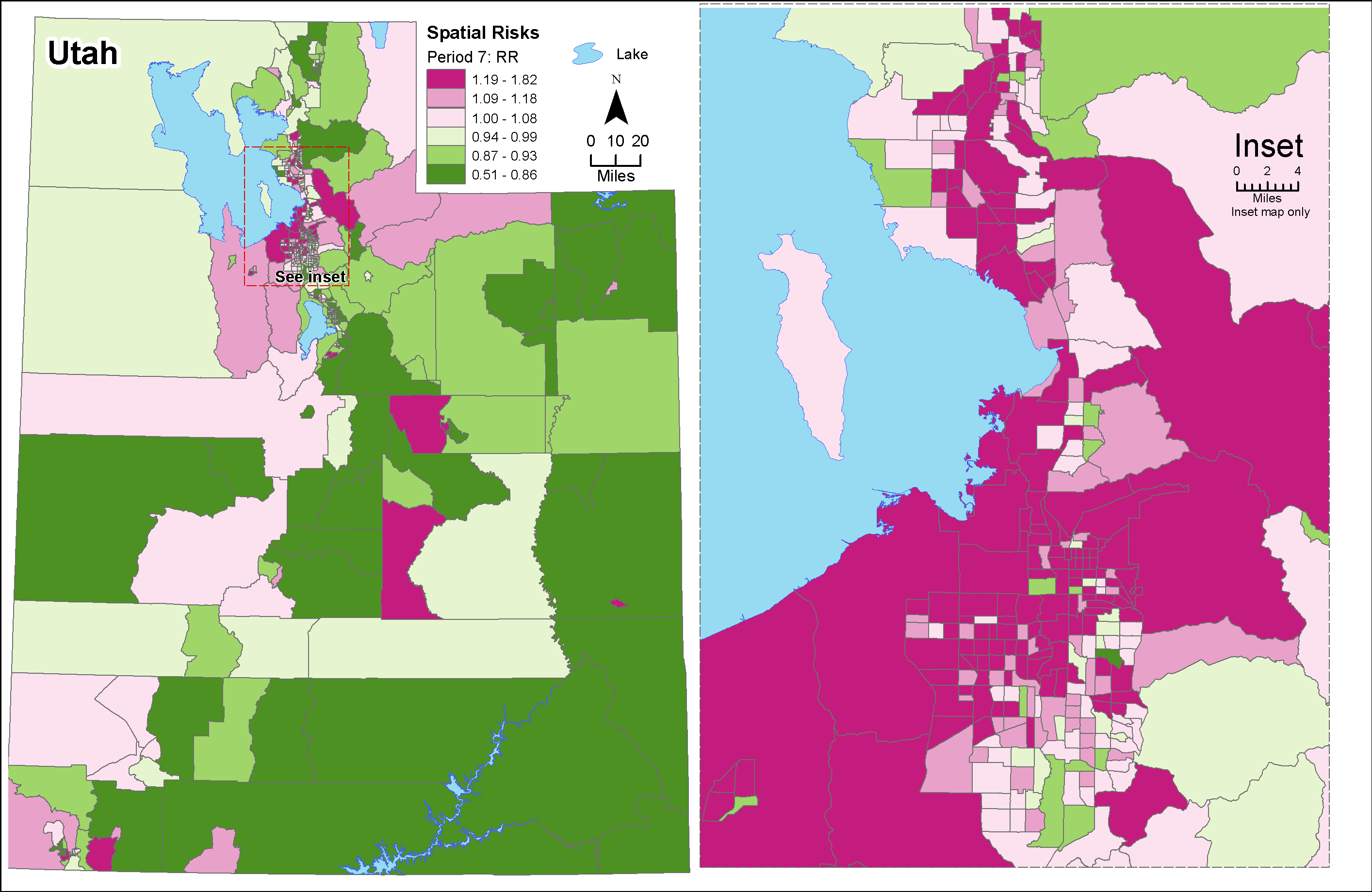

Supplement: Additional file 7 — Figure S7: Posterior medians of relative risks for 1997-2000 (period 7), both genders. Figure S7 displays the posterior medians of relative risks ρi7 for 1997-2000, using the spatio-temporal model. [file 1476-072X-10-16-S7.PNG]

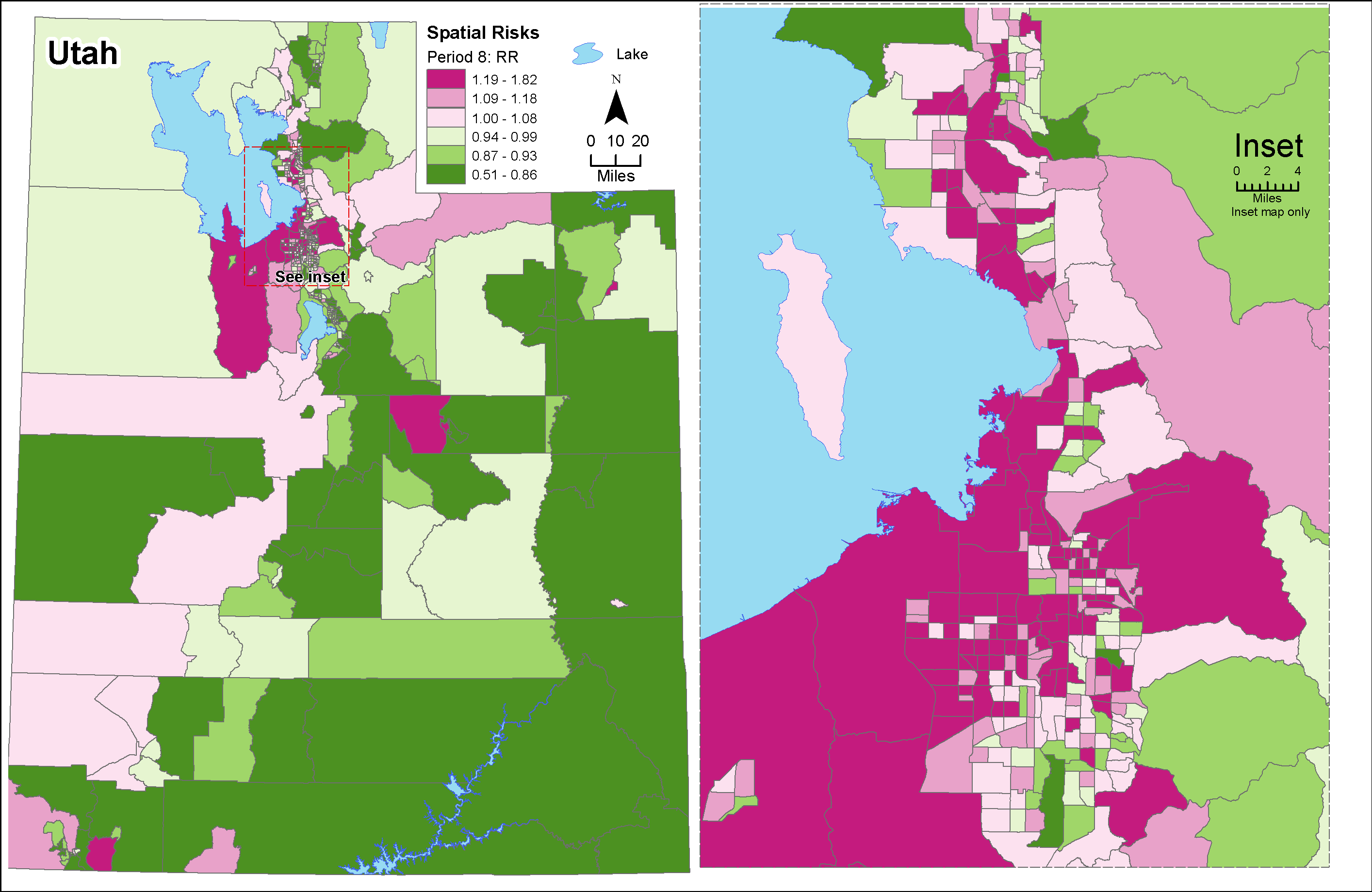

Supplement: Additional file 8 — Figure S8: Posterior medians of relative risks for 2001-2004 (period 8), both genders. Figure S8 displays the posterior medians of relative risks ρi8 for 2001-2004, using the spatio-temporal model. [file 1476-072X-10-16-S8.PNG]
